# First Application of the Orbeye™ 4K 3D Exoscope in Recurrent Papillary Thyroid Cancer Surgery

**DOI:** 10.3390/jcm12072492

**Published:** 2023-03-25

**Authors:** Michele D’Ambra, Anna Tedesco, Biancamaria Iacone, Umberto Bracale, Francesco Corcione, Roberto Peltrini

**Affiliations:** 1 Department of Public Health, Federico II University Hospital, 80131 Naples, Italy; 2 Department of Advanced Biomedical Sciences, Federico II University Hospital, 80131 Naples, Italy

**Keywords:** exoscope 4K 3D, Orbeye, thyroid surgery, papillary thyroid cancer, parathyroids

## Abstract

Reoperation for recurrent papillary thyroid cancer (RPTC) is much more complex than primary surgery is, with a higher rate of complications. We describe, for the first time, the use of the Orbeye™ surgical microscope/exoscope for the treatment of RPTC with lymphadenectomy. This system offers 4K, three-dimensional magnified and illuminated imaging without the need for eyepieces. Magnification of the field of view facilitates a more precise dissection, preserving the anatomical structure. Currently, the Orbeye™ is regularly used in neurosurgery; however, its potential in conventional open surgery has not yet been fully exploited. Owing to its magnification capacity, the Orbeye™ exoscope is a valuable tool to help surgeons identify and preserve the integrity of the recurrent laryngeal nerves and parathyroids during thyroid surgery.

## 1. Introduction

Papillary thyroid carcinoma (PTC) accounts for 75% of all thyroid cancers in the US [1]. The primary treatment for PTC is a total thyroidectomy. Cervical lymph node dissection is performed when it is indicated by clinical or ultrasonographic findings. The rate of PTC recurrence ranges from 10% to 30% [2]. When surgery is indicated for PTC recurrence, an increased risk of recurrent laryngeal nerve injury and temporary or permanent hypoparathyroidism can affect the postoperative outcomes, despite many efforts having been made over time to prevent nerve damage [3,4].

Since its introduction in the 1960s, the operating microscope (OM) has remained the gold standard for microsurgical operations because of it enables surgeons to manipulate structures with a diameter of less than 1 mm, such as nerves and vessels. However, the drawbacks of this system include the frequent need for repositioning and the fatigue associated with looking through the eyepieces for long periods of time. In the last few years, various exoscopes have been developed, such as Vitom^®^ 3D (Karl Storz, Tuttlingen, Germany), Orbeye™ (Olympus, Tokyo, Japan), and Modus VTM (Synaptive Medical, Toronto, ON, Canada). The Orbeye™ surgical microscope consists of two Sony 4K Exmor RTM CMOS image sensors and provides a high-sensitivity, low-noise, and wide-colour-range image, which is displayed on a 55-inch monitor and offers 4K three-dimensional (3D) magnified and illuminated imaging without the need for eyepieces. The operator visualises the surgical field using 3D glasses. Orbeye™ also has a semi-robotic flexible arm, which connects an LED light source to an ultrafast image processor. In addition to white-light imaging, the 4K 3D orbital camera system offers three different visualisation modes, which aim to improve the surgical workflow. Among these, Among these, the near-infrared (NIR) intrinsic autofluorescence of parathyroids facilitates their identification and the surgical dissection. Moreover, the infrared imaging mode provides bright 4K 3D intraoperative indocyanine green (ICG) fluorescence that allows fluorescence-guided magnification surgery to be performed.

Currently, the Orbeye™ surgical microscope is mainly used in neurosurgery, where it has proven to be a viable alternative to a standard OM [5]. The commonly cited benefits, which OM lacks, include superior ergonomics, comfort, and a wider field of vision.

We describe, for the first time, the use of the Orbeye™ for the treatment of recurrent papillary thyroid carcinoma (RPTC) with lymphadenectomy.

## 2. Case Presentation

The patient was a 20-year-old Caucasian male without comorbidities. Eight years prior, he had undergone a screening ultrasound that showed a nodule of 7 mm in the right lobe of his thyroid. Fine needle cytology was performed on this nodule, resulting in a classification of TIR5 (consistent with malignancy). In a different hospital, the patient underwent a total thyroidectomy with lymphadenectomy of the central compartment because of an intraoperative finding of suspicious lymph nodes. Pathology revealed papillary thyroid carcinoma with metastases in 3 of the removed lymph nodes (pT1a pN1a). After surgery, the patient underwent radio-metabolic therapy with 50 mCi of 131I, with evidence of uptake in the anterior region of the neck on post-dose scintigraphy. At the 6-year follow-up, laboratory tests showed detectable levels of thyroglobulin (Tg; 0.84 ng/mL). Thyroid ultrasound showed a rounded hypoechogenic formation (0.7 × 0.4 × 0.4 cm in size) in the right thyroid bed, a hypoechoic nodular area with hyperechoic shoots (0.5 × 1.2 × 1.6 cm in size) in the left thyroid bed, and a small hypoechoic area (0.2 × 0.4 cm in size) in the isthmus, while pericentromeric lymphadenopathies were present in the lateral cervical left bed. A Thyrogen^®^ test was performed, which revealed Tg levels of 4.70 ng/mL. Laboratory tests showed the following results: TSH 2.121 µU/mL, FT3 3.9 pg/mL, FT4 1.23 ng/dL, Tg 0.86 ng/mL, and AbTg < 0.9 IU/mL. Fine needle aspiration resulted in a classification of “TIR5, suspected recurrent RPTC” [6]. Reoperative surgery for RPTC recurrence and lymph node dissection was planned. Preoperative laryngoscopy was performed before surgery.

**Figure 1:**
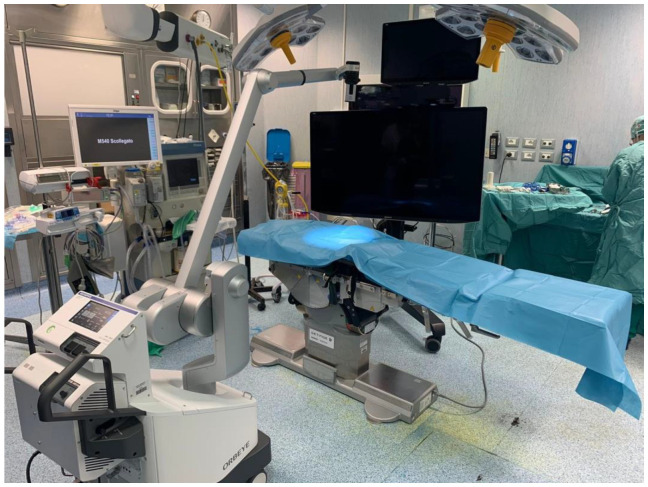
Orbeye exoscope 4K 3D and operating room setting.

**Figure 2:**
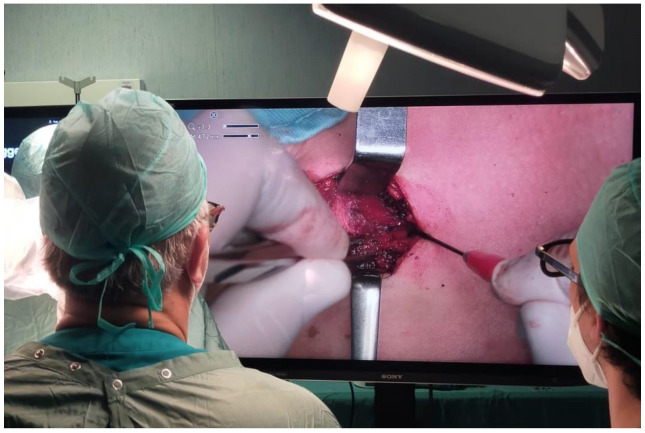
Intraoperative view of surgical field.

Orbeye™ was placed behind the main surgeon, and the semi-robotic arm was passed over his left shoulder (Figure 1). A sterile cover was placed over the semi-robotic arm so that the main surgeon could easily move the camera. The 55-inch monitor was positioned in front of the main surgeon on the other side of the table. An additional 32-inch screen was placed in front of the assistant surgeon to ensure adequate viewing. Another assistant surgeon positioned at the head of the patient for the duration of the surgery used the main screen. All surgeons wore 3D glasses (Figure 2).

**Figure 3:**
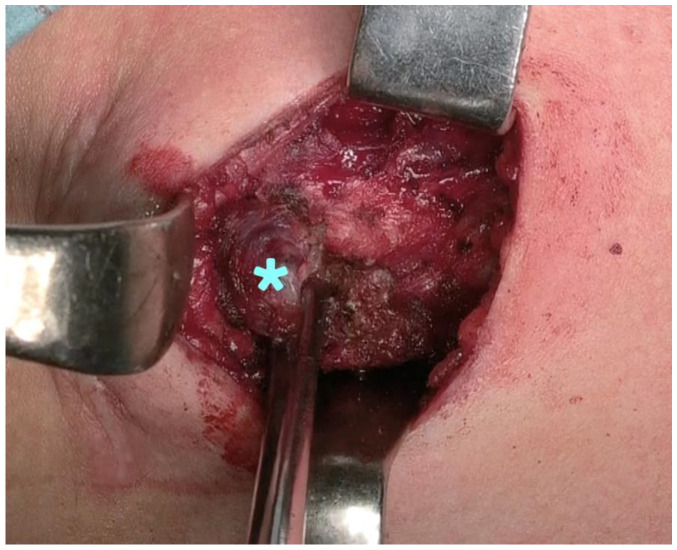
Residual thyroid tissue (*).

Access to the thyroid bed was obtained through mini-cervicotomy. Orbeye™ was used when we were accessing the thyroid bed. The magnification of the image allowed us to identify the residual thyroid tissue (Figure 3), excluding the involvement of the recurrent nerve in the lesion. Intermittent intraoperative neuromonitoring enabled the identification of the nerve. In this way, the dissection was conducted away from the nerve region, avoiding potential injury.

**Figure 4:**
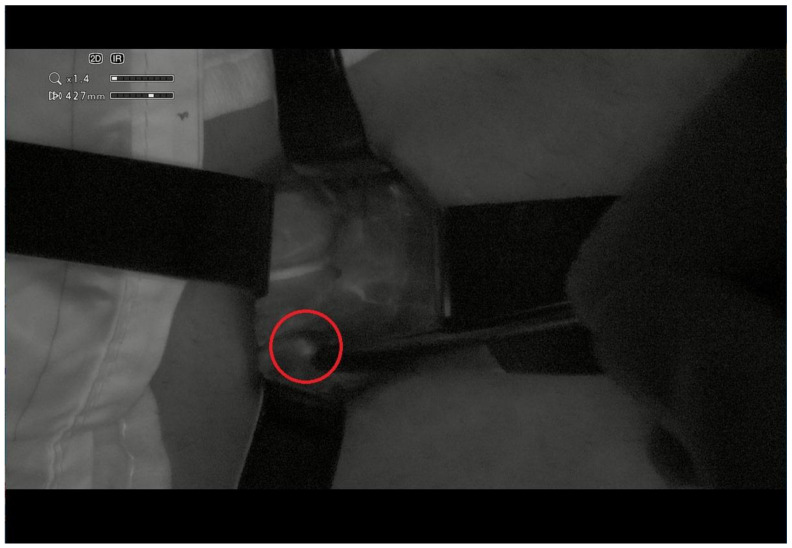
Use of NIR and ICG for visualization of the upper parathyroid (red circle).

We used NIR filters with ICG to highlight the parathyroids both before and at the end of the surgery to avoid the risk of postoperative hypocalcaemia (Figure 4) [7]. Drainage was not used.

No dysphonia or signs of hypocalcaemia were observed during the recovery period. The patient was discharged on the first postoperative day. Histological examination of the 2.5 × 1.5 cm specimen confirmed malignant RPTC with one metastatic lymph node of the central compartment (1.6 × 0.5 cm). Eight months after surgery, the Tg level was 0.8 ng/mL, and ultrasonography showed a hypoechoic nodular area of 0.5 × 1.2 × 1.7 cm. Molecular testing showed V600E-mutated BRAF.

## 3. Discussion

We report, for the first time, the use of the Orbeye™ exoscope for thyroid surgery in a case of RPTC. Magnification of the field of view allows a more precise dissection, preserving the anatomical structure.

Currently, the Orbeye™ is regularly used in neurosurgery; however, its potential in conventional open surgery has not yet been fully exploited. We had the opportunity to test Orbeye™ during different procedures. The first procedure performed was an inguinal hernia repair. Subsequently, pancreaticoduodenectomy, subtotal gastrectomy, the removal of three para-aortic masses, and Ivor Lewis esophagectomy were performed. Orbeye™ was used in several specific surgical steps in which magnification was required, such as for hepatic–jejunal and Wirsung–jejunal anastomosis or D2 lymph node dissection during gastrectomy [8].

Reoperation for RPTC is more complex than primary surgery is. Scar tissue and the modified anatomy of the surgical field can make the identification and preservation of recurrent laryngeal nerves and parathyroid glands more difficult. The incidence of permanent recurrent laryngeal nerve paralysis after reoperative thyroid surgery ranges from 1% to 12%. Similarly, the incidence of temporary hypoparathyroidism ranges from 0.3% to 13% [9].

New approaches for thyroid surgery have been developed over the years. These include robotic surgery with a transaxillary approach, which has been successful in Eastern countries. Evidence supports robotic thyroidectomy as a feasible and safe approach that is aesthetically superior to open surgery. However, no significant differences were identified between robotic and open thyroidectomies in terms of transient and permanent recurrent laryngeal nerve injury, permanent hypoparathyroidism, or haemorrhage [10]. Endoscopic surgery with the transoral approach is considered to be an alternative to anterior cervical incision. The advantages are the concealment of the incision within the floor of the mouth without significantly increasing the amount of dissection required and providing access to both sides of the neck. Although transoral endoscopic thyroid surgery provides remarkably direct and minimally invasive entry to the thyroid gland, there is a high rate of reported complications. Furthermore, the current technology limits surgeons to non-wristed instruments and requires manual control of the endoscope during dissection. Some of these limitations can be overcome with the use of robotic surgery, which provides high-resolution 3D vision and uses wristed instruments, tremor filtration, and precise robotic movement, which allows a degree of control and accuracy that are currently not available with endoscopic equipment [11]. Nevertheless, to date, the anterior approach with mini-cervicotomy remains the most widespread one because of the short operation time required and the low rate of complications, at the expense of a concealed scar.

Several therapeutic alternatives to surgery have been developed over time for treating both benign and malignant thyroid conditions. They consist in ultrasound (US)-guided procedures, such as radiofrequency, laser, microwave, high-intensity focused ultrasound (HIFU), and alcohol (ethanol) ablation. An international multidisciplinary consensus statement suggests using these percutaneous techniques as a first-line alternative to surgery for patients with benign thyroid nodules and for patients unfit or refusing surgery with primary papillary microcarcinoma or recurrent papillary thyroid carcinoma [12]. In particular, percutaneous laser ablation has gained popularity for the treatment of papillary thyroid microcarcinoma due to its safety, minimal trauma, rapid postoperative recovery, limited impact on thyroid function, and low recurrence rate in the long-term follow-up [13]. In addition, active surveillance is currently considered to be a viable option for papillary microcarcinoma. In most cases, it has a favourable impact on the patient’s quality of life and costs. Although the evidence refers mostly to Eastern populations, a limited but encouraging experience has been provided by Western countries [14].

Open video-assisted surgery has previously been used to perform thyroid surgery elsewhere [15]. Compared to the Vitom^®^ 3D exoscope, the Orbeye™ exoscope has a larger screen. More importantly, it is equipped with a semi-robotic arm that can be used with a sterile cover to ensure that the main surgeon can move the camera without the need for a joystick, managing the angle, distance from the operating field, and filter used. The ability to easily rotate the camera allows the best angle for visualisation of the operating field to be obtained, without the need to tilt the operating table.

The Orbeye™ exoscope presents clear advantages as an optical tool in performing conventional open surgery, owing to the improved magnification of the optical view, which provides high-definition 4K and 3D surgical images. The Orbeye™ exoscope has been proposed as a favourable alternative to the standard OM because of the high-quality image produced with adequate magnification and good depth of view [16]. Its major advantage is its ability to provide a wide field of view with a good depth of field compared to those of conventional OM. Despite their unquestionable advantages, standard OMs have several disadvantages. In particular, continuous repositioning is required when a wide surgical field is needed. Moreover, the surgeon must continually look through the eyepieces, which can lead to fatigue. Another advantage of Orbeye™ is the possibility of viewing the surgical field from various angles; this cannot be achieved with a traditional OM because its small and flexible optical unit cannot facilitate multiple surgical positions without compromising posture or creating positional discomfort. The Orbeye™ system also allows surgeons to have the same surgical vision using two different monitors. Finally, the Orbeye™ system is a valid tool for the training of young surgeons, allowing all the participants to visualise the operating field and the manoeuvres performed by the senior surgeon. Regarding the learning curve, the plateau performance and learning rate of ORBEYE are significantly noninferior to those of the microscope in a preclinical grape dissection task [17]. The use of the Orbeye™ exoscope can allow the surgeon to better visualise vascular and neural structures and may reduce the rate of complications.

## 4. Conclusions

Owing to its magnification capacity, the Orbeye™ exoscope is a valuable tool to help surgeons identify and preserve the integrity of the recurrent laryngeal nerves and parathyroids during thyroid surgery. Further studies are required to improve and standardise its use.

## Data Availability

Not applicable.
